# Acute kidney injury in hospitalized children with proteinuria: A multicenter retrospective analysis

**DOI:** 10.1371/journal.pone.0298463

**Published:** 2024-03-21

**Authors:** Katelyn H. Baggett, Tomas Manghi, Vonn Walter, Neal J. Thomas, Michael A. Freeman, Conrad Krawiec

**Affiliations:** 1 Pennsylvania State University College of Medicine, Hershey, Pennsylvania, United States of America; 2 Pediatric Critical Care Medicine, Department of Pediatrics, Penn State Hershey Children’s Hospital, Hershey, Pennsylvania, United States of America; 3 Department of Public Health Sciences, Pennsylvania State University College of Medicine, Hershey, Pennsylvania, United States of America; 4 Pediatric Nephrology and Hypertension, Department of Pediatrics, Penn State Hershey Children’s Hospital, Hershey, Pennsylvania, United States of America; 5 Department of Humanities, Pennsylvania State University College of Medicine, Hershey, Pennsylvania, United States of America; Indiana University School of Medicine, UNITED STATES

## Abstract

**Background and objective:**

Acute kidney injury (AKI) is a common complication in hospitalized pediatric patients. Previous studies focused on adults found that proteinuria detected during an admission urinalysis is fit to serve as an indicator for AKI and associated clinical outcomes. The objective of this study is to evaluate if proteinuria on the first day of hospital services in hospitalized children is associated with AKI, need for renal replacement therapy, shock and/or antibiotic use, critical care services, and all-cause mortality at 30 days, hypothesizing that it is associated with these outcomes.

**Methods:**

This is a retrospective cohort study using TriNetX electronic health record data of patients 2 to 18 years of age who underwent urinalysis laboratory testing on hospital admission, had three subsequent days of hospital or critical care services billing codes and creatinine laboratory values, and no pre-existing renal-related complex chronic condition. This study evaluated for the frequency, odds, and severity of AKI as defined by Kidney Disease: Improving Global Outcomes modified criteria and assessed for associated clinical outcomes.

**Results:**

This study included 971 pediatric subjects [435 (44.7%) with proteinuria]. Proteinuria on the first day of hospital services was associated with an increased odds for higher severity AKI on any day of hospitalization (odds ratio [OR] 2.41, CI 1.8–3.23, p<0.001), need for renal replacement therapy (OR 4.58, CI 1.69–12.4, p = 0.001), shock and/or antibiotic use (OR 1.34, CI 1.03–1.75, p = 0.033), and all-cause mortality at 30 days post-admission (OR 10.0, CI 1.25–80.5, p = 0.013).

**Conclusion:**

Children with proteinuria on the first day of hospital care services may have an increased odds of higher severity AKI, need for renal replacement therapy, shock and/or antibiotic use, and all-cause mortality at 30 days post-admission, with no significant association found for critical care services, mechanical intubation, or inotrope or vasopressor use.

## Introduction

Acute kidney injury (AKI) is a common complication in hospitalized pediatric patients, with a reported incidence rate of 30–50% in critically ill hospitalized children [1, 2]. Pediatric patients diagnosed with AKI have an in-hospital mortality rate six times that of hospitalized children with normal renal function, a significantly increased length of stay, and an increased odds of progression to chronic kidney disease (CKD) 5 years post-discharge [2, 3]. Thus, prompt recognition and treatment of AKI is necessary to improve clinical outcomes in hospitalized children. Findings of proteinuria on admission are often considered of unclear significance and as a transient elevation related to acute illness [4]. We sought to suggest whether findings of proteinuria on hospital admission may be associated with increased odds of AKI and poor clinical outcomes within the general population of hospitalized children.

The development of AKI has a multifactorial pathogenesis, ranging from a state of renal hypoperfusion (e.g. due to cardiac dysfunction, severe dehydration, significant blood loss, and sepsis), direct renal injury (e.g. exposure to nephrotoxic agents), or due to primary renal disease [1, 5, 6]. Clinically, AKI is defined by The Kidney Disease: Improving Global Outcomes (KDIGO) and several other practice guidelines as an increase in serum creatinine by ≥0.3 mg/dL (≥26.5 μmol/L) within 48 h, to ≥1.5 times baseline within the prior 7 days, or a urine volume of <0.5 mL/kg/h for 6 hours [7, 8].

Yet, there are several limitations of creatinine and urine output as disease markers for AKI in critically ill children [9]. Due to these shortcomings, previous studies focused on adults found that proteinuria detected during an admission urinalysis is fit to serve as an indicator for AKI and associated poor clinical outcomes in the setting of critical illness [10, 11]. This association, however, has yet to be explored in the pediatric population. If proteinuria is found to be associated with increased odds of AKI in hospitalized children, early interventions can potentially be implemented to minimize kidney damage and reduced morbidity and mortality. AKI in children is commonly secondary to an inflammatory systemic response, such as during infection, leading to glomerular injury and endothelial dysfunction and thus an increase in the permeability of albumin. If an association is found to exist between proteinuria and AKI, the detection of proteinuria on an admission urinalysis may indicate an increased odds for AKI and support prompt care escalation. Urinalysis may serve as a cost-effective, readily available, and rapid method to suggest odds and severity of acute kidney injury and clinical outcomes in hospitalized children.

The objective of this study is to evaluate whether proteinuria on the first day of hospital care services is associated with increased odds of higher severity AKI and subsequent clinical outcomes to support the goals of prompt diagnosis and care escalation for children hospitalized for any indication. We hypothesize that hospitalized children with proteinuria on the first day of hospital care services are at increased odds for AKI of higher severity, critical care service requirements, need for renal replacement therapy, and have an increased rate of all-cause mortality at 30 days post-admission.

## Methods

### Ethics statement

Our study was approved with exemption by the Institutional Review Board at the Pennsylvania State University (study number 20794) and received a waiver of informed consent. This is a retrospective cohort study using TriNetX electronic health record (EHR) data, a global, federated research network that provides access to anonymized EHR data from approximately 54 participating health care organizations (HCOs) largely within the United States. TriNetX, LLC is compliant with the Health Insurance Portability and Accountability Act (HIPAA), the United States federal law that protects the privacy and security of patient data. The data provided by TriNetX is de-identified on the basis of the de-identification standard defined in section §164.514(a) of the HIPAA Privacy Rule and is maintaied by an Information Security Management System to protect patient data according to HIPAA Security Rule requirements. The process by which datasets are de-identified is attested to through a formal determination by a qualified expert, as defined in section §164.514(b) of the HIPAA Privacy Rule. Since data from TriNetX is de-deidentified and because no protected health information is received by the user, authors did not have access to information that could identify individual participants or HCOs during or after data collection. EHR data elements were provided by TriNetX on 9/15/2022.

### Study design and data collection

**Fig 1:**
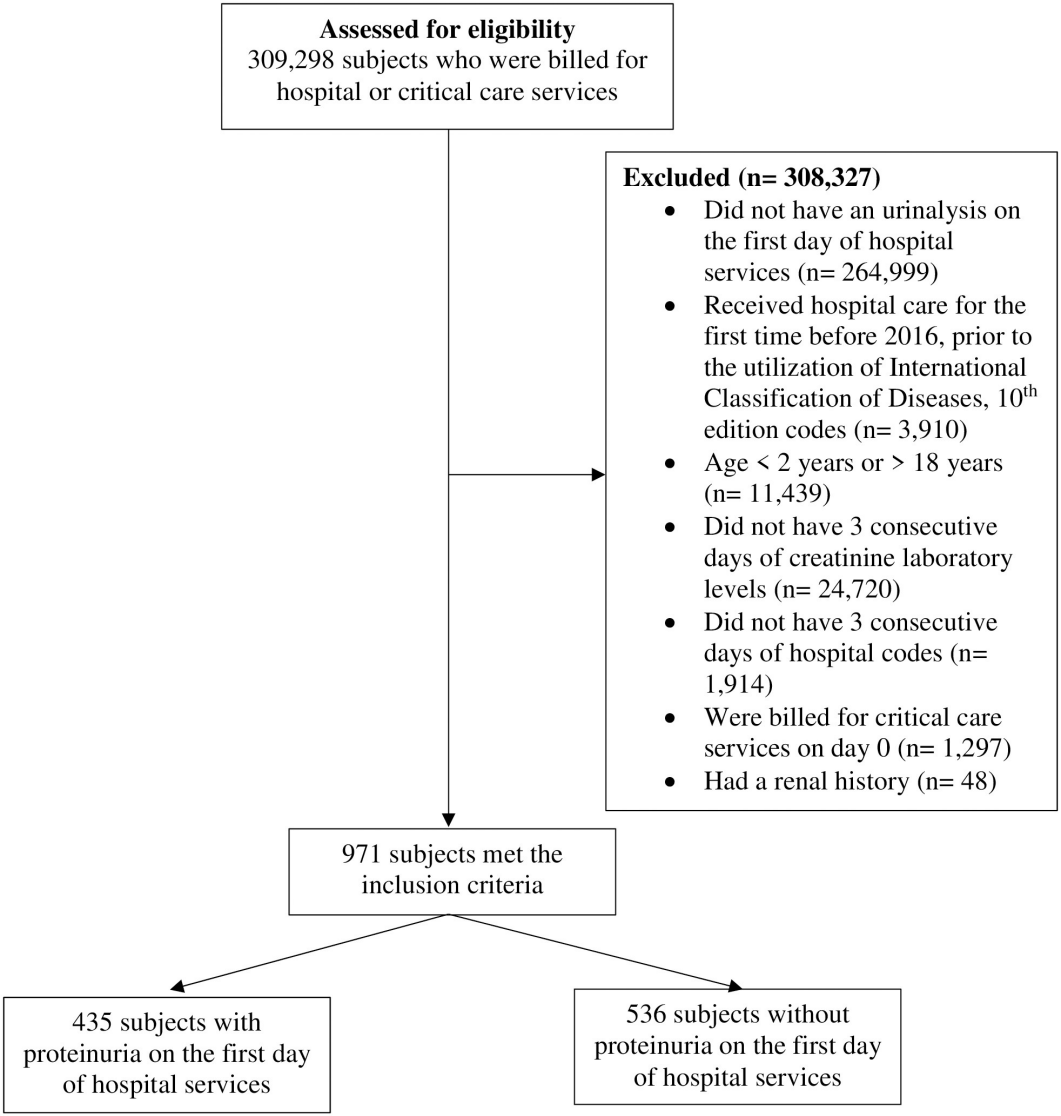
Flowchart of subject eligibility according to inclusion and exclusion criteria.

As outlined in Fig 1, TriNetX provided a de-identified dataset of electronic medical records of 309,298 subjects who were billed for hospital or critical care services from 54 HCOs. Of these, we included subjects that met the following inclusion and exclusion criteria: (1) underwent urinalysis or urine dipstick laboratory testing on day 0 of hospital services; (2) received hospital care for the first time in their database history after 2016; (3) were 2–18 years of age on day 0 of hospital services; (4) had 3 consecutive days of creatinine measurements to determine the odds of AKI; (5) were initially billed for hospital services and had 3 subsequent days of hospital or critical care service billing codes to evaluate for associated clinical outcomes; (6) had no pre-existing renal-related complex clinical condition as defined by the pediatric complex condition classification system’s catalog of renal and urologic ICD-9 and ICD-10 diagnostic codes [12]. Inclusion criteria of children with 3 days of hospitalization and creatinine laboratory values was strategically chosen to determine the odds of developing AKI at any time within the first three days of hospitalization and other associated clinical outcomes in this cohort of hospitalized children. Hospital admission was defined as the first time a subject was coded for hospital or critical care services after 2016, as this was when hospital administrative databases began to utilize International Classification of Diseases, 10^th^ edition codes (ICD-10) [13].

On 9/15/2022, we analyzed the following using the described dataset: demographic data (age, sex, race, ethnicity), ICD-9 and ICD-10 diagnostic codes, presence of any complex clinical condition as defined by the pediatric complex chronic conditions classification system [12] prior to the first day of hospital billing, creatinine laboratory values, indicators of critical illness [requirements for critical care services, renal procedures defined as either hemodialysis or continuous renal replacement therapy (CRRT), or mechanical ventilation], medication use (inotropes, vasopressors, antibiotics, or NSAIDs), and all-cause mortality. Race and ethnicity were included in this analysis to examine the presence of proteinuria in hospitalized patients among the different groups and as a surrogate for socioeconomic disparities that exists between groups. All clinical outcome variables were measured within 30 days from the first day of hospital services and are representative of the first hospitalization or readmission within a 30-day window.

Proteinuria on the first day of hospital care services was classified as either a negative or positive value. Referencing work by Neyra et al. within the adult population, a positive urinalysis for proteinuria was defined as a text value of “positive” or a numeric value of greater than 15.0 mg/dL, including trace proteinuria measured either by quantitative urinalysis or urine dipstick [10]. Utilizing this definition, we split the cohort into two groups, subjects with proteinuria (“positive” and/or greater than 15.0 mg/dL) and those without proteinuria (“negative” and/or less than 15.0 mg/dL). AKI was defined utilizing a modified approach to the KDIGO criteria. Baseline creatinine levels were identified from 14 days to 365 days prior to the initial creatinine level obtained on the first day of hospital care services. Because baseline creatinine levels on the first day of hospital care services were not available for all subjects within the database, we also calculated the baseline creatinine level based on age (<5 years = 0.3 mg/dL, 5–12 years = 0.5 mg/dL, ≥12 years = 0.7 mg/dL). Baseline creatinine values for subjects without a recorded baseline were chosen based off of the work done by Mah et al. where the same limitations of absent baseline creatinine values were present within their database [14]. These values were utilized to determine the odds of developing AKI at any time within the first three days of hospitalization according to the modified Kidney Disease: Improving Global Outcomes (KDIGO) Cr-based definition: KDIGO divides AKI into: Stage 1: Cr increase to 1.5–1.9 times baseline; Stage 2: Cr increase to 2–2.9 times baseline; Stage 3: Cr increase to ≥3 times baseline. Subjects with Stage 0 were considered having no AKI, while subjects with Stage 3 were considered having developed severe AKI. In this study and similar to studies within the adult population, only the SCr criterion for AKI was used given the lack of urine output data and concern for inaccurate documentation of urine output in the EHR [10, 15]. We based this definition on previously performed database studies where baseline creatinine was not available and urine output was not recorded requiring an estimation of baseline creatinine based on age [14].

ICD-9 and ICD-10 diagnostic codes were examined to suggest diagnoses that had an increased odds of presenting with proteinuria on the first day of hospital care services. Shock was inclusive of “hypovolemic shock”, “cardiogenic shock”, “severe sepsis with septic shock”, “other shock”, “shock, unspecified’, and “other shock without mention of trauma”. As some instances of sepsis were expected to have not been coded for, we sought to further identify children who may have been treated for sepsis through a code that included either a ICD-9 or ICD-10 diagnostic code for shock and/or any of the following antibiotics commonly used in sepsis: ampicillin, cefepime, cefotaxime, ceftriaxone, clindamycin, doxycycline, ertapenem, gentamicin, linezolid, meropenem, metronidazole, piperacillin, sulbactam, tazobactam, or vancomycin. NSAID exposure was analyzed between each group to further suggest the clinical status of children with proteinuria on the first day of hospital care services and served as a potential confounding variable.

For patient privacy, exact death dates were not provided by the TriNetX database, only the month and year. To meet the definition of death within 30 days, we first added 30 days to the date when the subject was first reported to have received hospital care services. If the month and year of the death date provided matched this calculation, the patient met the definition of death within 30 days.

### Statistical analysis

Summary counts for the frequency of AKI as defined by KDIGO criteria, shock and/or antibiotic use, requirements for renal replacement therapy, mechanical ventilation, inotropic/vasopressor support, and all-cause mortality at 30 days post-admission in the proteinuria and no proteinuria groups were computed and summarized in contingency tables. Given the small counts for the race categories “American Indian or Alaska Native” and “Native Hawaiian or Other Pacific Islander”, these groups were combined with “Asian” to form an “Other” race group when analyzing the demographic data. Subjects with “Unknown” ethnicity were not included in the analysis of the ethnicity data. Fisher’s exact test was applied when analyzing contingency tables involving unordered factors, and the Wilcoxon rank sum test was used when analyzing quantitative values. Two-sided p-values less than 0.05 were considered statistically significant. The epitools R package [16] was employed to compute odds ratios and 95% confidence intervals using the “Wald” method when analyzing the contingency table data. The DescTools R package [17] was used to apply the Cochran-Armitage trend test for contingency tables of KDIGO stage. Separate multivariable logistic regression models were fit using the variables of either shock/antibiotic use or death as the binary response. Wald 95% confidence intervals were computed for all parameter estimates, and these were exponentiated to calculate confidence intervals for the adjusted odds ratios. Race was dichotomized as “White” or “Non-White” in the logistic regression models. All analyses were performed using R 4.2.2 [18].

## Results

### Patient characteristics

In this study, 971 subjects met the inclusion criteria. Of these, 435 (44.7%) subjects had proteinuria on the first day of hospital care services. The mean admission age of the total population was 9.7 ± 5.0 years [9.7 ± 5.0 years or 9.8 ± 4.9 years in the groups without and with proteinuria, respectively]. There were no statistically significant differences of pre-existing complex clinical conditions in the proteinuria compared to the no proteinuria groups, signifying a low likelihood of comorbidities complicating the presentation of each group. Age, sex, race, ethnicity, and pre-existing complex clinical conditions are summarized in Table 1.

**Table 1:** Characteristics of pediatric subjects.

Characteristic	Total (n = 971)	No Proteinuria (n = 536)	Proteinuria (n = 435)	p-value (p<0.05)
Age (mean ± SD), years	9.7±5.0	9.7 ± 5.0	9.8 ± 4.9	**0.71**
Female, n (%)	468 (48.2%)	272 (50.7%)	196 (45.1%)	0.08
Male, n (%)	503 (51.8%)	264 (49.3%)	239 (54.9%)	0.08
**Race/Ethnicity, n (%)**				0.003
American Indian or Alaska Native	3 (0.3%)	2 (0.4%)	1 (0.2%)	
Asian	25 (2.6%)	12 (2.2%)	13 (3.0%)	
Black or African American	177 (18.2%)	76 (14.2%)	101 (23.2%)	
Native Hawaiian or Other Pacific Islander	3 (0.3%)	3 (0.6%)	0	
White	628 (64.7%)	360 (67.2%)	268 (61.6%)	
Hispanic or Latino Ethnicity	135 (13.9%)	93 (17.4%)	42 (9.7%)	
**Pre-existing Complex Clinical Conditions, n (%)**				
Cardiovascular	70 (7.2%)	37 (6.9%)	33 (7.6%)	0.68
Gastrointestinal	51 (5.3%)	28 (5.2%)	23 (5.3%)	0.96
Hematologic or Immunologic	78 (8.0%)	46 (8.6%)	32 (7.4%)	0.48
Malignancy	84 (8.7%)	54 (10.1%)	30 (6.9%)	0.08
Metabolic	61 (6.3%)	35 (6.5%)	26 (6.0%)	0.72
Neurologic and Neuromuscular	56 (5.8%)	35 (6.5%)	21 (4.8%)	0.26
Respiratory	24 (2.5%)	15 (2.8%)	9 (2.1%)	0.47

### International Classification of Diseases, 10th edition diagnostic code categories of pediatric subjects with and without proteinuria on the first day of hospital services

The diagnostic codes most commonly associated with proteinuria were diseases of the genitourinary system (p<0.001), infectious and parasitic diseases (p = 0.007), diseases of the musculoskeletal system and connective tissue (p<0.001), and diseases of the respiratory system (p = 0.006). [Table 2]

**Table 2:** International Classification of Diseases, 10^th^ edition diagnostic code categories.

Diagnostic Code, n (%)	Total (n = 971)	No Proteinuria (n = 536)	Proteinuria (n = 435)	p-value (p<0.05)
Diseases of the genitourinary system	338 (34.8%)	127 (23.7%)	211 (48.5%)	< 0.001
Certain infectious and parasitic diseases	275 (28.3%)	133 (24.8%)	142 (32.6%)	0.007
Diseases of the musculoskeletal system and connective tissue	300 (30.9%)	142 (26.5%)	158 (36.3%)	< 0.001
Diseases of the respiratory system	304 (31.3%)	148 (27.6%)	156 (35.9%)	0.006

### Frequency of acute kidney injury according to stage and day of hospitalization

Children with proteinuria on the first day of hospital care services were at increased odds for AKI as compared to children without proteinuria (odds ratio [OR] 2.41, CI 1.8–3.23, p<0.001). Children with proteinuria on the first day of hospital services were more likely to have higher severity AKI as classified by KDIGO, on any day of hospitalization (p<0.001). [Table 3]

**Table 3:** Frequency of AKI by stage and day of hospitalization.

Measure of AKI, n (%)	Total (n = 971)	No Proteinuria (n = 536)	Proteinuria (n = 435)	Odds Ratio (95% CI)	Cochran-Armitage trend test
Frequency of Acute Kidney Injury Defined by KDIGO Stage	255 (26.3%)	100 (39.2%)	155 (60.8%)	2.41 (1.8–3.23)	< 0.001
**Day 0**					< 0.001
KDIGO Stage 1	89 (9.2%)	44 (49.4%)	45 (50.6%)		
KDIGO Stage 2	61 (6.3%)	18 (29.5%)	43 (70.5%)		
KDIGO Stage 3	72 (7.4%)	16 (22.2%)	56 (77.7%)		
**Day 1**					< 0.001
KDIGO Stage 1	65 (6.7%)	29 (44.6%)	36 (55.4%)		
KDIGO Stage 2	44 (4.5%)	13 (29.5%)	31 (70.5%)		
KDIGO Stage 3	70 (7.2%)	21 (30.0%)	49 (70.0%)		
**Day 2**					< 0.001
KDIGO Stage 1	55 (5.7%)	24 (43.6%)	31 (56.4%)		
KDIGO Stage 2	40 (4.1%)	16 (40.0%)	24 (60.0%)		
KDIGO Stage 3	61 (6.3%)	15 (24.6%)	46 (75.4%)		

### Indicators of critical illness and clinical outcomes of pediatric subjects

There was an increased odds of renal replacement therapy, including hemodialysis and CRRT, in the proteinuria group as compared to the no proteinuria group (OR 4.58, CI 1.69–12.4, p = 0.001). Children with proteinuria on the first day of hospital care services had an increased odds of a subsequent shock diagnosis and/or antibiotic use (OR 1.34, CI 1.03–1.74, p = 0.03) and an increased odds of all-cause mortality at 30 days post-admission (OR 10.0, CI 1.25–80.5, p = 0.013). There was no significant difference found for need of critical care services, mechanical ventilation, or inotrope or vasopressor use in children with proteinuria, as compared to children without proteinuria. Proteinuria on the first day of hospital care services and subsequent NSAID use was not determined to be associated with statistical significance. [Table 4]

**Table 4:** Indicators of critical illness and clinical outcomes.

Variable, n (%)	Total (n = 971)	No Proteinuria (n = 536)	Proteinuria (n = 435)	Odds Ratio (95% CI)	p-value (<0.05)
Frequency of Renal Replacement Therapy	20 (2.1%)	4 (0.7%)	16 (3.7%)	4.58 (1.69–12.4)	0.001
Shock/ Antibiotic Use	365 (37.6%)	185 (34.5%)	180 (41.4%)	1.34 (1.03–1.74)	0.03
Death at 30 days post-admission	9 (0.9%)	1 (0.2%)	8 (1.8%)	10 (1.25–80.50)	0.01
Critical Care Medicine Code	76 (7.8%)	39 (7.3%)	37 (8.5%)	1.2 (0.82–1.75)	0.38
Mechanical Ventilation (any, first 3 days)	41 (4.2%)	28 (5.2%)	13 (3.0%)	0.67 (0.36–1.25)	0.22
Inotrope or Vasopressor Use (any, first 3 days)	81 (8.3%)	48 (9.0%)	33 (7.6%)	0.98 (0.70–1.39)	0.93

### Multivariate analysis of the association of antibiotic use and mortality with presence of age, sex, race, and presence of proteinuria on the first day of hospitalization

In multivariate analysis, there was an increased odds of shock and/or antibiotic use in the proteinuria group as compared to the no proteinuria group (OR 1.34, CI 1.03–1.76, p = 0.03). Similarly, in multivariate analysis, children with proteinuria on the first day of hospital care services had an increased odds of all-cause mortality at 30 days post-admission (OR 11.6, CI 1.35–100, p = 0.03). [Table 5]

**Table 5:** Multivariate analysis of the association of antibiotic use and mortality with prescnce of age, sex, race, and presence of proteinuria on the first day of hospitalization.

	Shock/Antibiotic Use		Mortality	
	OR (95% CI)	p-value (<0.05)	OR (95% CI)	p-value (<0.05)
**Age**	0.97 (0.94, 0.991)	0.01	0.76 (0.60, 0.957)	0.02
**Sex (Reference: Male)**	0.88 (0.67,1.16)	0.36	3.49 (0.62,19.7)	0.16
**Race (Reference: White)**	0.93 (0.71,1.24)	0.63	0.47 (0.084,2.66)	0.40
**Critical Care Services**	3 (2.02,4.44)	<0.001	26.8 (5.14,141)	<0.001
**Presence of Proteinuria**	1.34 (1.03,1.76)	0.03	11.6 (1.35,100)	0.03

## Discussion

This study aimed to determine the impact of proteinuria detected by the first admission urinalysis obtained in hospitalized pediatric patients. While the association between proteinuria and severe AKI is previously well-established in adults, this study explores whether this finding is relevant to the pediatric population and sought to determine if the presence of proteinuria correlates with the severity of illness and clinical outcomes [10]. This study’s main findings were that children with proteinuria on the first day of hospital care services may have an increased frequency of AKI defined by KDIGO criteria, with a greater odds of higher severity AKI on any day of hospitalization. Additionally, children with proteinuria had an increased odds of shock and/or antibiotic use and had an increased requirement for hemodialysis or CRRT. Despite children with proteinuria on the first day of hospital care services having an increased rate of all-cause mortality at 30 days post-admission, there was no significant association found for critical care medicine codes, mechanical intubation, or inotrope or vasopressor use, as compared to children without proteinuria on the first day of hospital care services. These findings may have significant implications in how clinicians approach the presence of proteinuria in hospitalized pediatric patients.

Given the significant impact of AKI on clinical outcomes, it is crucial to identify at-risk patients to support early intervention and treatment. There are, however, several limitations of current methods used to diagnose AKI as defined by KDIGO criteria. Serum creatinine can be falsely decreased in the setting of massive fluid resuscitation and is known to be a delayed manifestation of AKI with some estimates projecting a peak serum concentration at 72 hours post-kidney injury [19, 20]. Within the pediatric population, creatinine is limited as an indicator of AKI due to low baseline production of creatinine according to low muscle mass, a lack of a baseline creatinine level for comparison, and increased risk for malnutrition [9, 21]. Additionally, studies specific to the pediatric population suggest that heterogeneity in creatinine testing regimens can contribute to clinically significant variation in reported creatinine values [22, 23]. As male children have a faster rate of muscle mass development when compared to female children, creatinine reference ranges are suggested to be increasingly inaccurate during the adolescent period [24]. In regards to urine output, immediate changes may not be readily apparent and urine collection is susceptible to inaccuracies of measurement. Each of these factors may lead to delayed or missed diagnosis of AKI within the pediatric population and increase the risk of adverse clinical outcomes.

Proteinuria detected through urinalysis has been previously implicated as a risk factor for adverse renal-related outcomes [25]. In a retrospective observational study of critically ill septic patients, de novo proteinuria detected within the first 24 hours of admission was associated with the development of severe AKI [10]. During the era of the COVID-19 pandemic, another study suggested that findings of proteinuria on admission predicted future development of AKI during hospitalization, supporting the use of urinalysis as a risk-assessment tool in critically ill adults [15]. Our results expand upon these findings, though specific to the pediatric population, and reinforce the notion that the presence of proteinuria may suggest increased odds for severe AKI according to KDIGO staging.

Often, findings of proteinuria on admission are considered of unclear significance as a transient elevation related to acute illness. Further, previous reports suggest that proteinuria detected by urinalysis testing has a high incidence of false-positive test results when testing concentrated urine, which may erroneously decrease a clinician’s concern for this finding [4]. Though, it is important to note that the presence of measurable proteinuria on urinalysis in the setting of a concentrated specimen may itself be suggestive of a pre-renal physiology and relative decrease in renal perfusion, which in turn increases the risk of progression to more severe forms of AKI. Our findings of an association between proteinuria detected during an admission urinalysis and odds of greater severity AKI highlights the importance of further clinical investigation, repeat testing, and increased renal protective measures through avoidance of nephrotoxic medications. Increased suspicion for AKI may prompt the clinician to reconsider the primary diagnosis and facilitate early transfer to critical care services to improve clinical outcomes and reduce morbidity and mortality within the pediatric population.

Our findings also suggest that children with proteinuria on the first day of hospital care services may have an increased odds of shock and/or subsequent antibiotic use. In studies focused on adults diagnosed with septic shock, proteinuria is associated with a decreased rate of recovery from AKI at 30 days post-hospital discharge and increased critical illness scores [10]. The occurrence of proteinuria upon hospital admission in patients with sepsis is also correlated with an increased length of ICU stay and fewer ventilator-free and vasopressor-free days, serving to predict the need for highly intensive medical management [26]. Thus, the early detection and treatment of AKI is necessary to improve clinical outcomes in the critical care setting and prevent the use of nephrotoxic antibiotics, many of which are commonly used in sepsis.

Despite findings of an increased all-cause mortality at 30-days post-admission, this study did not identify an increased odds for critical care services, mechanical ventilation, or inotrope or vasopressor use within the proteinuria group. There are several explanations for this finding. First, it is possible that children with proteinuria received critical care services without being billed within the EHR. Second, it is plausible that the clinical severity of children presenting with proteinuria is underrecognized and undertreated with a lack of care escalation. Finally, the subject may have been referred to another center for critical care services.

This study has several limitations. As the objective of this study was to determine if the presence of proteinuria on admission was associated with increased odds and severity of AKI during the first three days of pediatric hospitalization, including the first day, it is important to note that we do not predict the subsequent development of AKI following the detection of proteinuria. As AKI may have been present for some subjects during hospital admission, we are unable to suggest a timeline between the detection of proteinuria on admission urinalysis and the onset of AKI. While it is possible that this study’s inclusion criteria of three consecutive days of hospitalization and creatinine laboratory values may have selected for children with more severe illness, this duration of hospitalization was specifically chosen in order to determine the odds for AKI and associated clinical outcomes. In regards to this study’s definition of AKI, while the inclusion criteria of three consecutive days of hospitalization and creatinine laboratory values excluded children who met the AKI diagnostic criteria of >1.5 times increase in creatinine over 7 days, this lengthened time of hospitalization markedly decreased our sample size and would have decreased the internal and external validity of the study. Because urine output is not reported in this dataset, we only were able to utilize creatinine as a measure of AKI in this population. Thus, without having urine output as part of the definition of AKI, some subjects with a normal reported creatinine possibly had a higher stage of AKI, given that creatinine rise may be a delayed manifestation of AKI. We also acknowledge the limitation of subjects having a one-time urinalysis performed on hospital admission, given the possibility of false-positives when evaluating for the presence of proteinuria with urinalysis. While this possibility cannot be ruled out, we attempted to mitigate the occurrence of false-positive test results by utilizing a large cohort. As this study was a retrospective design and limited to the healthcare organizations that participate in the TriNetX database, it is possible that not all EHR data was reported. Due to EHR database limitations, we were unable to identify the primary diagnosis of this patient population. This heterogeneity can potentially impact the generalizability of our findings. Future study may be needed for specific patient populations, such as pediatric patients with sepsis, to further suggest clinical outcomes specific to each diagnosis. Similar to studies within the adult population, we focused our analysis to the presence of proteinuria and did not evaluate the presence of other urinalysis components (i.e. erythrocytes). Because of this, we were unable to evaluate other potential causes for proteinuria according to other urinalysis parameters. Race and ethnicity were included in this analysis to examine the presence of proteinuria in hospitalized patients among the different groups. Due to the multicenter, de-identified nature of the database, it is unknown how race and/or ethnicity data were collected and whether they were self-reported. Due to the small sample size for children who received renal replacement therapy or had an increased rate of all-cause 30-day mortality, the confidence intervals of the multivariable logistic regression were wide, suggesting that while there may be a higher odds of proteinuria within these populations, the increase may be slight. As a result of the small counts for these variables, future study with an increased sample size is needed for further analysis. Because convenience sampling was utilized for this study, power analysis could not be performed.

## Conclusions

This study suggests that children with proteinuria on the first day of hospital care services may have an increased odds of higher severity AKI during hospitalization, shock and/or antibiotic use, need for renal replacement therapy, and all-cause mortality at 30 days post-admission. As there were no signs of increased critical care service requirements, illness severity may be underrecognized in hospitalized children with proteinuria. While further prospective study with an increased sample size is needed for in-depth analysis, proteinuria on admission urinalysis may suggest increased odds for AKI and poor clinical outcomes.
